# Pediatric imaging

**Published:** 2009-02

**Authors:** IK Indrajit

**Affiliations:** Department of Radiodiagnosis and Imaging, Army Hospital (Research and Referral), Delhi Cantt – 110 010, India

Few useful websites related to Pediatric Imaging are reviewed below:

1. **Virtual Pediatric Hospital** http://www.virtualpediatrichospital.org/ focuses on topics that covers imaging of various disease entities in pediatrics. Created by Donna. D'Alessandro and Michael D'Alessandro, the material is available “for use by residents, fellows, or attending physicians practicing pediatrics”. Some of the useful educative material comprises Correlapaedia - A Correlative Encyclopedia of Pediatric Imaging, Surgery and Pathology, ElectricAirway - Upper Airway Problems in Children, MetaTextbook of Pediatric Radiology - Imaging Appearances of Common Pediatric Diseases, Paediapaedia - An Imaging Encyclopedia of Pediatric Disease, Pediatric Jeopardy and Thoracopaedia - An Imaging Encyclopedia of Pediatric Thoracic Disease.
2. **PediatricEducation.org** is a “pediatric digital library and learning collaboratory intended to serve as a source of continuing pediatric education”. Available at http://www.pediatriceducation.org/, the educative material is divided into sections titled Learning Collaboratory, Cases by Disease or Symptom or Specialty or Age or Date. The site is curated by Donna D'Alessandro, M.D. and Michael D'Alessandro, M.D. There is a separate section on Reference Library and discussions.
3. **Pedrad.org** is presented from the Society for Pediatric Radiology at http://www.pedrad.org. This society is a “professional medical organization, committed to improving the health care of patients by providing excellence in diagnostic imaging and treatment of neonates, infants, children and adolescents.” The educative sections, featured in the website includes Case of the Month, Research and Education Foundation and Internet Links.
4. **Pedrad Info** at http://www.pedrad.info is a web portal focusing on pediatrics, pediatric radiology and pediatric surgery. The site is authored by Talanow R and Hirsch W, has many sections hosting a variety of features such as an Online Book, Discussion Forum, Most Interesting Cases and Case of the Day etc. Submit and publishing cases is allowed. Besides this, an educative ‘pediatric radiology case collection’ is also available.
5. **Picubook** at http://lib.cpums.edu.cn/jiepou/tupu/atlas/pedsccm.wustl.edu/All-Net/main.html is a website that focuses as an on-line resource for pediatric critical care. The site fully realizes the potential of web based learning in paediatrics. It has articles, videos, graphics, lectures, animation, slides, javascript calculators, innovative sections and NLM search threads.
6. **Scintigraphy of the Pediatric Skeleton** is available at http://www.medical-atlas.org/. The site has a search engine that will allow the user to find cases and/or images based on a free text search or a search by case for known diagnosis or a search for specific image characteristics. Searching using free text search, case search, image search and using the skeleton is possible. The development of this web site is linked to the publication of two atlases of Bone Scintigraphy in Paediatrics, produced under the auspices of the Paediatric Commitee of the European Association of Nuclear Medicine.
7. **Online Pediatric Handbook** at http://home.coqui.net/titolugo/handbook.htm comprises 11 chapters, each dealing with a specific topic. While the early chapters deal with head and neck topics, obstructive problems, the later chapters deal at length on Hernias and abdominal wall defect, gastrointestinal bleeding and pancreatic and biliary conditions. Separate sections on interesting topics are available towards the end concerning tumors, prenatal congenital malformations and pediatric laparoscopy.
8. **Plain Radiographic Diagnosis Of Congenital Heart Disease** is presented at http://www.bcm.edu/radiology/cases/pediatric/index.htm. The material authored by C McMahon and E Singleton, M.D. from Texas Children's Hospital, deals with the normal chest radiograph image and description and lists a set of conditions linked by a common radiological finding.
9. **Images in Pediatric Cardiology** is an online journal available at http://www.impaedcard.com/ The issues covering 1999 - 2008 contains topics covering Embryology, Endocarditis, Cardiomyopathies, Syndromes associated with congenital heart disease and a host of other topics.
10. **Pediatric Radiology Normal Measurements** is a handy reckoner, sourced from Department of Radiology, Oregon Health and Science University at http://www.ohsu.edu/radiology/teach/kojima/. Stemming from an initial scientific exhibit at the 1999 annual meeting of the Society for Pediatric Radiology, the material was originally authored by K Kojima MD, R Thomas MD, and P Silberberg MD. The web material enables radiologists to access normal measurements in categories such as Chest, Gastrointestinal, Endocrine, Renal, Musculoskeletal and Neuroradiology.

## Endpiece

RSNA education portal has an educative section titled “**From the Archives of the AFIP**”. The pediatric contents covering topics like Chest/Cardiac, Gastrointestinal, Genitourinary, Musculoskeletal, Neuroradiology Imaging is available at http://www.rsna.org/Education/archive/afip.cfm#pediatric.

**Radquiz** at http://www.radquiz.com/ is a gateway to Radiology resources, maintained by N Ahmad, M.D. The website has a host of links to a wide variety of educative sites available across the net. This site has links to different sections such as Neurology, GU, GI, Pediatric, Chest, Musculoskeletal, Sonography etc. The physics sections at http://www.radquiz.com/physics.htm is useful from the point of view of radiology students. An interesting section “Radiology online oral board case review” has teaching cases that are broadcast to participants in a live, real-time virtual meeting.

Moving to a different and an exclusive area in radiology, there are magazines offering latest information on imaging modalities as well as an insight into the ‘business’ of contemporary radiology practices. Few of these useful websites for radiologists and administrators include **Imaging Economics** at http://www.imagingeconomics.com/ is a “journal has provided a monthly, high-level forum to address the development, diffusion, acquisition, and utilization of imaging technology”. Interesting sections includes buyer's guide and resources.

**Imaging Management** at http://www.imagingmanagement.org/ focuses on “the technology advances, methods to improve the radiology services, best practice, better value-for-money investments, cost savings, imaging informatics and optimal patient and staff satisfaction”.

**Medical Imaging** at http://www.medicalimagingmag.com/about.asp is “a leading monthly news and business magazine for radiology and imaging professionals who are involved in selecting, managing, and supporting technology that is used in the medical-imaging department of a healthcare provider facility”.

**Diagnostic Imaging Magazine** at http://www.diagnosticimaging.com/home has many interesting sections like Net seminars, Imaging trends and Advances focus on technology, Daily news to name a few. A Topic Centers offers information on Cardiac imaging, CT colonography, Digital X-ray, Healthcare IT, Imaging center, Molecular imaging, MRI, Musculoskeletal, nuclear, PACS, Practice management, radiation Oncology, Residents, Software center, Teleradiology, Ultrasound, Women's imaging. Additionally, there are sections covering Newsletter Sign-Up, Radiology Education, CME and White Papers.

**Medical Imaging International at** http://www.medimaging.net/ is a journal focusing on the latest techniques in medical imaging. Sections cover clinical applications, scientific advances, new products, technical literature, and international events worldwide. “Subjects covered in every issue include: Radiology, Ultrasound, MR Imaging, Cardiovascular Imaging, Nuclear Medicine, Radiotherapy, and Administration”.

